# Boost und Hypofraktionierung beim DCIS

**DOI:** 10.1007/s00066-022-02016-y

**Published:** 2022-10-20

**Authors:** Jürgen Dunst, David Krug

**Affiliations:** Klinik für Strahlentherapie Kiel, UKSH, Kiel, Deutschland

## Hintergrund

Für Patientinnen mit DCIS ist die postoperative Radiotherapie nach der aktuellen S3-Leitlinie zwar kein obligater Bestandteil bei brusterhaltender Therapie, aber sie die beste Maßnahme zur Senkung des Rückfallrisikos und wird als individuelle Maßnahme empfohlen [5]. Der Stellenwert des Boosts ist, anders als bei invasiven Karzinomen, bislang unklar; in der Leitlinie wird sinngemäß nur die Strahlentherapie der ganzen Brust ohne Boost empfohlen [5]. Die jetzt publizierten Daten der BIG/TROG-Studie zeigen, dass auch beim DCIS ein Boost das Rückfallrisiko weiter senken kann [2].

## Patienten und Methodik

Die BIG-3-07/TROG-07.01-Studie ist eine randomisierte Therapiestudie, an der sich 136 Einrichtungen aus 11 Ländern beteiligten. Patientinnen mit Non-low-risk-DCIS und freien Resektionsrändern (> 1 mm) nach brusterhaltender Op. wurden randomisiert zwischen Ganzbrustbestrahlung ohne bzw. mit Boost. Eine zweite Randomisierung zur Fraktionierung (konventionelle Fraktionierung mit 50 Gy in 25 Fraktionen oder Hypofraktionierung mit 42,5 Gy in 16 Fraktionen) war möglich; alternativ konnten sich alle Zentren vor Studienbeginn für eines der beiden Fraktionierungsregime entscheiden und dann alle Patientinnen so behandeln. Der Boost wurde immer nach der Ganzbrustbestrahlung mit einer Dosis von 16 Gy in 8 Fraktionen appliziert. Non-low-risk-DCIS war definiert als das Vorliegen von mindestens einem der folgenden Risikofaktoren: Alter < 50 Jahre, palpabler Tumor/Symptome, Durchmesser ≥ 15 mm, Multifokalität, Kerngrad („intermediate/high nuclear grade“), zentrale Nekrose, Komedonekrose, freier Resektionsrand < 10 mm. Eine endokrine Therapie (mit Tamoxifen) war erlaubt. Als Studienhypothese wurde eine Verbesserung der In-Brust-Tumorkontrolle nach 5 Jahren durch den Boost von 93 auf 96 % (HR 0,56) angenommen.

## Ergebnisse

Zwischen Juni 2007 und Juni 2014 wurden 1608 Patientinnen in die Studie aufgenommen. Von den Patientinnen wurden 503 bezüglich Boost und Fraktionierung randomisiert, 581 wurden randomisiert für den Boost bei Vorauswahl für konventionelle Fraktionierung und 524 randomisiert bei Vorauswahl für Hypofraktionierung. Es zeigte sich ein signifikanter Vorteil durch den Boost im Hinblick auf die lokale Tumorkontrolle: Die Lokalrezidivrate in der Brust nach 5 Jahren betrug 7,3 % ohne Boost vs. 2,9 % mit Boost (HR 0,47, *p* = 0,00031). Insgesamt war der Effekt des Boosts also besser als erwartet (HR 0,47, die statistische Planung ging ja von 0,56 aus). Die verbesserte lokale Kontrolle war verbunden mit einem verbesserten krankheitsfreien Überleben nach 5 Jahren (89,6 % ohne Boost vs. 93,7 % mit Boost, HR 0,63, *p* = 0,004). Erwartungsgemäß bestand kein Effekt auf das Überleben (5-J-Überleben 98,2 % ohne Boost vs. 99,0 % mit Boost, HR 0,81, *p* = n. s.). In multivariater Analyse war der Boost der wichtigste Prognosefaktor für ein intramammäres Rezidiv; der einzige weitere Risikofaktor war die Tumorgröße.

Allerdings war der Boost auch mit signifikant mehr Nebenwirkungen verbunden. Relevante Spätfolgen, vor allem Brustschmerzen und Induration/Fibrose, waren zwar selten, aber die absolute Zunahme von Grad-2–4-Spätfolgen (plus 11 %) war höher als die Reduktion der Lokalrezidive (minus 3,4 %). Wenn man nur Spätfolgen von Grad 3–4 betrachtet (und Grad-2-Spätfolgen außer Acht lässt), sah das etwas günstiger aus (Zunahme etwa 0,5 %).

Es bestand kein signifikanter Einfluss der Fraktionierung auf die Ergebnisse. Bei Patientinnen, die bezüglich der Fraktionierung randomisiert wurden, betrug das 5‑J-DFS 90 % bei konventioneller Fraktionierung vs. 92,4 % bei Hypofraktionierung; in der Gesamtgruppe aller Patientinnen war das 5‑J-DFS 91,0 % bei konventioneller Fraktionierung vs. 92,4 % bei Hypofraktionierung.

## Schlussfolgerungen

Die Autoren folgern, dass erstens ein Boost auch beim DCIS die lokale Kontrolle optimiert und dass zweitens Hypofraktionierung auch beim DCIS sinnvoll ist.

## Kommentar

Die adjuvante Strahlentherapie nach brusterhaltender Op. ist auch beim DCIS die beste Maßnahme zur Senkung des Rückfallrisikos in der Brust. Die höchste Evidenz stammt aus der Metaanalyse der Early Breast Cancer Trialists’ Collaborative Group (EBCTCG) von 2010 [4]. In diese Metaanalyse gingen Daten aus vier randomisierten Studien mit 3729 Patientinnen ein. Die Bestrahlung der Brust (ein Boost war in diesen Studien nicht erlaubt bzw. nicht vorgesehen) reduzierte das Rückfallrisiko nach 10 Jahren von 28,1 auf 12,9 % (HR 0,46). Die wichtigste Studie der letzten Jahre war die RTOG-9804-Studie, die in der EBCTCG-Metaanalyse noch nicht berücksichtigt wurde und in der Patientinnen mit Low-risk-DCIS untersucht wurden [7, 8]. Die Strahlentherapie der Brust senkte das Rückfallrisiko zunächst sehr beeindruckend (HR 0,11) von 6,7 auf 0,9 % nach 7 Jahren [7]. In der kürzlich publizierten Langzeitauswertung nach 15 Jahren war der absolute Effekt zwar stabil mit einer Rezidivreduktion von 15,1 auf 7,1 % nach 15 Jahren, der relative Effekt nahm jedoch durch den verzögerten Anstieg der Rezidive im Bestrahlungsarm ab (HR 0,36; [8]). In der multivariaten Analyse war der Effekt der Strahlentherapie stärker als der Effekt von Tamoxifen (HR 0,34 für RT bzw. 0,44 für Tamoxifen; Tamoxifen war nicht obligat, aber erlaubt und war bei 69 % der Patientinnen in beiden Armen eingesetzt worden). Spättoxizitäten von Grad 3 oder höher waren in beiden Armen (mit und ohne RT) 4 %. Die Strahlentherapie der ganzen Brust ist also weiterhin das effektivste Verfahren zur Rezidivreduktion und sie ist (nach den Daten von RTOG 9804) sehr gut verträglich.

Der Stellenwert des Boosts war bisher aber schwieriger zu bewerten. Es gab bisher keine randomisierten Studien, und Metaanalysen aus retrospektiven Studien und großen Kohortenanalysen zeigten nur einen geringen oder keinen Effekt (HR etwa 0,7 bis 0,9) oder einen Effekt nur bei positiven Rändern [9, 10]. Daneben gibt es auch theoretische Argumente gegen den Boost; das Rezidivmuster beim DCIS ist nämlich weniger eindeutig als bei invasiven Karzinomen, bei denen viele Rezidive „echte“ Lokalrezidive sind, während es beim DCIS überwiegend um neue Tumoren geht.

Dass die BIG- und TROG-Studiengruppen also eine randomisierte Studie gestartet und außerdem einen ziemlich starken Effekt (HR 0,56 als Studienhypothese; stärker als in bekannten retrospektiven Analysen) angenommen haben, ist mutig und bewundernswert. Dass diese Studie dann auch noch einen stärkeren Effekt als erwartet zeigt, ist überraschend und beeindruckend. Das unterstreicht den Stellenwert der Strahlentherapie beim DCIS. Die Bedeutung der Studie wird ferner unterstrichen durch die Tatsache, dass sie im selben Heft mit einem Kommentar gewürdigt wurde [3]. Die Kommentatoren sind allerdings etwas zurückhaltender in der Bewertung und sehen die Daten zur Hypofraktionierung als wichtigstes Studienergebnis. Aus unserer Sicht ergeben sich für den praktischen Alltag folgende Konsequenzen:

- Die Strahlentherapie ist bei der brusterhaltenden Therapie die beste Maßnahme zur Senkung des Rückfallrisikos in der Brust; das gilt auch für das DCIS.
- Der hier beobachtete relative Effekt des Boosts ist vergleichbar mit dem relativen Effekt bei invasiven Karzinomen, wie er in der EORTC-Studie beobachtet wurde. Indirekt betätigen diese aktuellen Daten daher auch die schon sehr alten Boost-Daten der EORTC-Studie und der Lyon-Studie [1, 11]. Die absolute Risikoreduktion fällt aber geringer aus als bei der Ganzbrustbestrahlung und in den älteren Boost-Studien beim invasiven Karzinom. Eine Risikosenkung um 4 % entspricht einer „number needed to treat“ von 25 zur Verhinderung eines Lokalrezidivs bzw. ca. 50 zur Verhinderung eines invasiven Rezidivs.
- Ganz nebenwirkungsfrei war der Boost in dieser Studie nicht, insbesondere waren die hier applizierten 16 Gy mit signifikant mehr Spätfolgen assoziiert, wenngleich bezüglich der Grad-3–4-Fibrosen etwas geringer als in der EORTC-Studie [12]. Die Kommentatoren empfehlen eine individuelle Nutzen-Risiko-Bewertung und die Einbeziehung der Patientin in die Entscheidung im Sinne eines „shared decision making“.
- Aus heutiger Sicht ist ein sequenzieller Boost mit 16 Gy sicher nicht mehr das Optimum. Es gibt gute Argumente für einen simultanen Boost, aber bisher noch keine belastbaren Daten. Die ersten Ergebnisse der RTOG-1005-Studie (sequenzieller vs. simultan-integrierter Boost bei invasiven Karzinomen) werden in diesem Jahr auf dem ASTRO-Kongress vorgestellt werden.
- Die wichtigste Botschaft der Studie (und in diesem Punkt stimmen wir den Kommentatoren eindeutig zu) ist sicher die Gleichwertigkeit der Hypofraktionierung auch beim DCIS. Das war aus Subgruppenanalysen und retrospektiven Daten zwar anzunehmen, wurde jetzt aber erstmals mit höchster Evidenz belegt [6]. Und das sollte auch in der täglichen Praxis konsequent umgesetzt werden!
- Die Daten sprechen insgesamt dafür, dass man die bei invasiven Karzinomen eingesetzten Dosierungs- und Fraktionierungskonzepte auch beim DCIS verwenden kann und sollte. Dazu wird es hoffentlich demnächst auch entsprechende Empfehlungen der Fachgesellschaften geben.

## Fazit

Strahlentherapie ist auch beim DCIS sehr effektiv und gut verträglich. Ein Boost ist auch beim DCIS mit Risikofaktoren sinnvoll und sollte individuell abgewogen werden. Und: Hypofraktionierung ist auch beim DCIS die Fraktionierung der ersten Wahl.

*Jürgen Dunst & David Krug, Kiel*

## References

[1] Bartelink H, Maingon P, Poortmans P (2015). Whole-breast irradiation with or without a boost for patients treated with breast-conserving surgery for early breast cancer: 20-year follow-up of a randomised phase 3 trial. Lancet Oncol.

[2] Chua BH, Link EK, Kunkler IH (2022). Radiation doses and fractionation schedules in non-low-risk ductal carcinoma in situ in the breast (BIG 3–07/TROG 07.01): a randomised, factorial, multicentre, open-label, phase 3 study. Lancet.

[3] Coles CE, Chatterjee S, Jagsi R, Kirby AM (2022). Breast radiotherapy for ductal carcinoma in situ: could less be more?. Lancet.

[4] Early Breast Cancer Trialists’ Collaborative Group (2010). Overview of the randomised trials of radiotherapy in ductal carcinoma in situ of the breast. J Natl Cancer Inst Monogr.

[5] Interdisziplinäre S3-Leitlinie für die Früherkennung, Diagnostik, Therapie und Nachsorge des Mammakarzinoms, Version 4.4. von Juni 2021. https://www.awmf.org/uploads/tx_szleitlinien/032-045OLl_S3_Mammakarzinom_2021-07.pdf. Zugriff 9. Sept. 2022

[6] Marta GN, Riera R, Pacheco RL (2022). Moderately hypofractionated post-operative radiation therapy for breast cancer: systematic review and meta-analysis of randomized clinical trials. Breast.

[7] McCormick B, Winter KA, Woodward W (2021). Randomized phase III trial evaluating radiation following surgical excision for good-risk ductal carcinoma in situ: long-term report from NRG oncology/RTOG 9804. J Clin Oncol.

[8] McCormick B, Winter K, Hudis C (2015). RTOG 9804: a prospective randomized trial for good-risk ductal carcinoma in situ comparing radiotherapy with observation. J Clin Oncol.

[9] Moran MS, Zhao Y, Ma S (2017). Association of radiotherapy boost for ductal carcinoma in situ with local control after whole-breast radiotherapy. JAMA Oncol.

[10] Nilsson C, Valachis A (2015). The role of boost and hypofractionation as adjuvant radiotherapy in patients with DCIS: a meta-analysis of observational studies. Radiother Oncol.

[11] Romestaing P, Lehingue Y, Carrie C (1997). Role of a 10-Gy boost in the conservative treatment of early breast cancer: results of a randomized clinical trial in Lyon, France. J Clin Oncol.

[12] Vrieling C, Collette L, Fourquet A (1999). The influence of the boost in breast-conserving therapy on cosmetic outcome in the EORTC “boost versus no boost” trial. Int J Radiat Oncol Biol Phys.

